# Molecular characterisation of protist parasites in human-habituated mountain gorillas (*Gorilla beringei beringei*), humans and livestock, from Bwindi impenetrable National Park, Uganda

**DOI:** 10.1186/s13071-017-2283-5

**Published:** 2017-07-18

**Authors:** Matthew J. Nolan, Melisa Unger, Yuen-Ting Yeap, Emma Rogers, Ilary Millet, Kimberley Harman, Mark Fox, Gladys Kalema-Zikusoka, Damer P. Blake

**Affiliations:** 1 Department of Pathobiology and Population Sciences, Royal Veterinary College, University of London, Hatfield, UK; 2 Conservation through Public Health, Plot 3 Mapera Lane, Uringi Crescent, Entebbe, Uganda

**Keywords:** Zoonosis, Zooanthroponosis, Infectious disease, *Cryptosporidium*, *Giardia*, *Entamoeba*

## Abstract

**Background:**

Over 60 % of human emerging infectious diseases are zoonotic, and there is growing evidence of the zooanthroponotic transmission of diseases from humans to livestock and wildlife species, with major implications for public health, economics, and conservation. Zooanthroponoses are of relevance to critically endangered species; amongst these is the mountain gorilla (*Gorilla beringei beringei*) of Uganda. Here, we assess the occurrence of *Cryptosporidium*, *Cyclospora*, *Giardia*, and *Entamoeba* infecting mountain gorillas in the Bwindi Impenetrable National Park (BINP), Uganda, using molecular methods. We also assess the occurrence of these parasites in humans and livestock species living in overlapping/adjacent geographical regions.

**Results:**

Diagnostic PCR detected *Cryptosporidium parvum* in one sample from a mountain gorilla (IIdA23G2) and one from a goat (based on SSU). *Cryptosporidium* was not detected in humans or cattle. *Cyclospora* was not detected in any of the samples analysed. *Giardia* was identified in three human and two cattle samples, which were linked to assemblage A, B and E of *G*. *duodenalis*. Sequences defined as belonging to the genus *Entamoeba* were identified in all host groups. Of the 86 sequence types characterised, one, seven and two have been recorded previously to represent genotypes of *Cryptosporidium*, *Giardia*, and *Entamoeba*, respectively, from humans, other mammals, and water sources globally.

**Conclusions:**

This study provides a snapshot of the occurrence and genetic make-up of selected protists in mammals in and around BINP. The genetic analyses indicated that 54.6% of the 203 samples analysed contained parasites that matched species, genotypes, or genetic assemblages found globally. Seventy-six new sequence records were identified here for the first time. As nothing is known about the zoonotic/zooanthroponotic potential of the corresponding parasites, future work should focus on wider epidemiological investigations together with continued surveillance of all parasites in humans, other mammals, the environment, and water in this highly impoverished area.

**Electronic supplementary material:**

The online version of this article (doi:10.1186/s13071-017-2283-5) contains supplementary material, which is available to authorized users.

## Background

Zoonoses are often considered as infectious diseases (IDs) acquired by humans via (in)direct contact with animal species that act as carriers of the infective agents. However, there is increasing evidence for the transmission of IDs from humans to livestock and wildlife species [1]. Here, etiological agents of concern include viruses, bacteria and protists. For instance, the diarrhoeal disease caused by *Cryptosporidium parvum*, that is transmitted from cattle to humans and *vice versa*, is responsible for economic losses in livestock animals, particularly calves, linked to mortality, morbidity, and subsequent human (re)infections as a consequence of poor hygiene [2]. Wild animals are also at risk from diseases originating in humans, e.g. human Ebola virus [3] or *Yersinia pestis* [4], or domesticated animals, e.g. canine distemper virus (morbillivirus) [5]. Given the ‘threatened’ status of many wildlife species which are already at risk from anthropogenic activities (i.e. illegal hunting, habitat modification), the increased threat of disease transmission from humans and livestock animals, and subsequent changes to host-parasite dynamics because of smaller habitat ranges imposes unnecessary risks on their continued survival.

The mountain gorilla (*Gorilla beringei beringei*) is critically endangered [6], and lives in two isolated regions, the Bwindi Impenetrable National Park (BINP) in Uganda and the Virunga Volcanoes Conservation Range, bounded by Uganda, Rwanda and the Democratic Republic of Congo [7, 8]. In 1993, several mountain gorilla groups were habituated to humans to promote wildlife tourism and behavioural research. In addition to the increased human contact as a direct result of these activities, the habituation process has led to gorillas venturing outside protected regions to forage. The areas surrounding BINP are subject to extreme ecological imbalances with 300–500 people/km^2^ and high numbers of livestock, both with low-quality health services. As a result, gorilla conservation is now also threatened by the increased risk of disease transmission from humans and livestock. Precautions are required to avoid interspecies transmission of ‘novel’ pathogens [9, 10].

The accurate identification of parasites from animals and environmental samples (i.e. soil, water) underpins a holistic approach to disease control. Given the limitations of conventional microscopic methods and host origin to the specific diagnosis of many parasites (i.e. *Cryptosporidium*, *Giardia*, *Cyclospora* and *Entamoeba* [11–14]), tools based on PCR have been used to characterise samples. Genetic characterisation has benefitted our understanding of epidemiology, host and geographical ranges, and assessing the risk infected hosts pose as reservoirs for interspecies infection. Despite the availability of these molecular techniques, substantial gaps in our knowledge remain. Here, we carried out a molecular study of protists infecting mountain gorillas, livestock and humans, from sites in and around BINP. We used PCR and targeted amplicon sequencing to detect and characterise parasites. The genotypes defined here were compared with published resources to provide insights into the epidemiology of disease in and around BINP and the potential for interspecies transmission.

## Methods

### Bwindi impenetrable National Park, Uganda

The Bwindi Impenetrable National Park (1°4′50″S, 29°39′41″E), Uganda, covers 32,092 ha and is located on the eastern edge of the Albertine Rift Valley, sharing a border with the protected Sarambwe forest in the Democratic Republic of Congo. The National Park was created in 1991 to protect the critically endangered mountain gorilla. The Park experiences a wet and mild climate with a mean temperature range of 11–23 °C, no real dry season, and provides diverse habitats ranging from 1160 to 2706 m in altitude. BINP is renowned as a biodiversity hotspot (see http://whc.unesco.org/en/list/682), and is home to ~340 of the critically endangered mountain gorilla. Surrounded by one of the poorest and most densely populated rural areas in Uganda with more than 300–500 people/km^2^, BINP has little possibility of a buffer zone with the surrounding agricultural landscape.

### Sample collection

A total of 203 faecal deposits from *Gorilla beringei beringei* (mountain gorilla; *n* = 68), *Bos taurus* (cattle; *n* = 45), *Capri hircus* (goat; *n* = 57), and *Homo sapiens* (human; *n* = 33) were collected from locations in and around BINP during May to June 2015 (see Table 1). All faecal samples were transported to the Conservation through Public Health Laboratory (BINP, Uganda) immediately after collection and fixed with 96% ethanol in a 2:1 ratio of ethanol to faeces. All samples were sent to the Royal Veterinary College for molecular analysis.

**Table 1:** The total numbers of each host species sampled at each site, together with the seven *Gorilla beringei beringei* Groups sampled from three separate regions of BINP, and geographical coordinates

Site	Geographical coordinates	Host species				Total
		*Gorilla beringei beringei*	*Bos taurus*	*Capra hircus*	*Homo sapiens*	
Aidah-Rugira	00°58′26.0″S, 029°36′44.0″E			6		6
Buhoma	00°58′34.1″S, 029°38′00.5″E	13 fr. Group Rushegura				13
	00°59′37.9″S, 029°37′47.2″E	10 fr. Group Mubare				10
	00°58′15.4″S, 029°36′48.2″E			16		16
	00°58′06.4″S, 029°37′00.0″E				3	3
Bujengwe Parish	00°55′53.5″S, 029°40′33.4″E				27	27
Kanyamisinga	00°57′26.0″S, 029°36′46.5″E		15	6		21
Karangara Nyakahanga	00°58′06.4″S, 029°37′00.0″E				1	1
Kayonza Mukono	00°58′06.4″S, 029°37′00.0″E				1	1
Kihembe Nabirehe	00°58′06.4″S, 029°37′00.0″E				1	1
Mukono, Church of Uganda	00°58′07.9″S, 029°37′09.2″E		12			12
	00°58′26.9″S, 029°37′20.0″E			19		19
Murutojo	00°58′25.9″S, 029°41′7.7″E	14 fr. Group Habinyanja				14
Nkwenda	00°58′27.5″S, 029°36′55.0″E		18			18
	00°58′49.7″S, 029°36′50.7″E			10		10
Ruhija (East)	01°04′31.4″S, 029°46′59.3″E	8 fr. Group Bitukura				8
	01°03′48.6″S, 029°46′46.2″E	8 fr. Group Research/Kyiaguliro				8
South	01°05′39.1″S, 029°39′01.5″E	8 fr. Group Nkuringo				8
	01°03′22.4″S, 029°37′25.8″E	7 fr. Group Bushaho				7
Total		68	45	57	33	203

From seven habituated gorilla groups from three different sectors of the park, samples were collected from night-nests each morning and were less than eight hours old. Duplicated samples were avoided by sampling each group only once and taking just one sample per nest. Livestock faecal deposits were collected on privately owned farms bordering BINP. Samples were collected either directly from the rectum or the ground; care was taken to collect only those parts of the faeces not in contact with the environment. Humans that inhabited villages surrounding BINP and had frequent interactions with free-ranging gorillas provided faecal samples.

### Isolation of genomic DNA

Total genomic DNA was isolated from each faecal sample using a QIAamp Fast DNA Stool Mini Kit (Qiagen, Hilden, Germany). In brief, a 500–800 μl aliquot of each sample was centrifuged at 10,000×*g*/1 m, the supernatant removed, 1 ml of phosphate buffered saline added to the pellet (0.2–0.3 g) and the samples mixed by a vortex mixer for 10 s. Following further centrifugation at 10,000×*g*/1 m, the supernatant was removed, glass beads (0.4–0.6 mm diameter) (Sigma-Aldrich, St Louis, USA) to the equivalent of 0.5 volume of the faecal pellet added, and the sample homogenised using a BeadBeater (30,000× oscillations/min for 30 s). Total genomic DNA was then extracted as per the manufacturer’s instructions and stored at -20 °C.

### PCR amplification, gel electrophoresis, sequencing, and phylogenetic analysis

Nested PCR was used to amplify total genomic DNA. For the specific identification of *Cyclospora*, *Cryptosporidium* and *Entamoeba*, a portion of the small subunit of the ribosomal DNA (SSU) was amplified (~500 bp, ~240 bp, and 382–429 bp, respectively). The genotypic and subgenotypic classification of *Cryptosporidium* was achieved using part of the 60 kDa glycoprotein gene (*gp60*; 250–350 bp). For the genetic characterisation of *Giardia* (to the level of assemblage), portions of the triosephosphate isomerase (*tpi*; ~ 530 bp), glutamate dehydrogenase (*gdh*; ~530 bp), beta-giardin (*bg*; ~511 bp), and SSU (~292 bp) genes were amplified. PCR was carried out in a volume of 50 μl containing ~200 ng of DNA template, 50 pmol of each primer, 25 μl of 2× MyTaq™ Mix (Bioline, Taunton, USA), and made up to 50 μl with DNase/RNase free H_2_O (ThermoFisher Scientific, Hemel Hempstead, UK). Table 2 lists the primers and cycling protocol used to amplify each gene.

**Table 2:** PCR primers and cycling protocols to amplify target sequences from *Cryptosporidium*, *Cyclospora*, *Giardia* and *Entamoeba*

Parasite	PCR target	Size (bp)	Primer	Reference	Cycling protocol	Reference
*Cyclospora*	SSU	1000	ExCycF (forward: 5′-AATGTAAAACCCTTCCAGAGTAAC-3′)	[90]	94 °C/ 5 min, followed by 35 cycles of 94 °C/ 45 s, 55 °C/ 45 s and 72 °C/ 1 min, with a final extension of 72 °C/ 10 min	[91]
			ExCycR (reverse: 5′-GCAATAATCTATCCCCATCACG-3′)			
		500	NesCycF (forward: 5′-AATTCCAGCTCCAATAGTGTAT-3′)		Secondary amplification was achieved employing identical PCR conditions to those used in the primary PCR	
			NesCycR (reverse: 5′-CAGGAGAAGCCAAGGTAGGCRTTT-3′)			
*Cryptosporidium*	SSU	824–864	XF2 (forward: 5′-GGAAGGGTTGTATTTATTAGATAAAG-3′)	[92]	94 °C/ 5 min, followed by 35 cycles of 94 °C/ 45 s, 60 °C/ 45 s and 72 °C/ 1 min, with a final extension of 72 °C/ 10 min	[92]
			XR2 (reverse: 5′-AAGGAGTAAGGAACAACCTCCA-3′)			
		298	18SiF (forward: 5′-AGTGACAAGAAATAACAATACAGG-3′)	[93]	94 °C/ 5 min, followed by 35 cycles of 94 °C/ 45 s, 50 °C/ 45 s and 72 °C/ 1 min, with a final extension of 72 °C/ 10 min	[93]
			18SiR (reverse: 5′-CCTGCTTTAAGCACTCTAATTTTC-3′)			
	*gp60*	1000	AL3531 (forward: 5′-ATAGTCTCCGCTGTATTC-3′)	[94]	94 °C/ 5 min, followed by 35 cycles of 94 °C/ 45 s, 50 °C/ 45 s and 72 °C/ 1 min, with a final extension of 72 °C/ 10 min	[95]
			AL3535 (reverse: 5′-GGAAGGAACGATGTATCT-3′)	[96]		
		457	AL3532 (forward: 5′-TCCGCTGTATTCTCAGCC-3′)	[94]	Secondary amplification was achieved employing identical PCR conditions to those used in the primary PCR	
			AL3533 (reverse: 5′-GAGATATATCTTGGTGCG-3′)			
*Giardia*	*tpi*	605	AL3543 (forward: 5′-AAATTATGCCTGCTCGTCG-3′)	[84]	94 °C/ 5 min, followed by 35 cycles of 94 °C/ 45 s, 50 °C/ 45 s and 72 °C/ 1 min, with a final extension of 72 °C/ 10 min	[84]
			AL3546 (reverse: 5′-CAAACCTTTTCCGCAAACC-3′)			
		530	AL3544 (forward: 5′-CCCTTCATCGGTGGTAACTT-3′)		Secondary amplification was achieved employing identical PCR conditions to those used in the primary PCR	
			AL3545 (reverse: 5′-GTGGCCACCACTCCCGTGCC-3′)			
	*bg*	753	G7 (forward: 5′-AAGCCCGACGACCTCACCCGCAGTGC-3′)	[63]	94 °C/ 5 min, followed by 35 cycles of 94 °C/ 30 s, 65 °C/ 30 s and 72 °C/ 1 min, with a final extension of 72 °C/ 7 min	[63]
			G759 (reverse: 5′-GAGGCCGCCCTGGATCTTCGAGACGAC-3′)			
		511	bgiF (forward: 5′-GAACGAACGAGATCGAGGTCCG-3′)	[97]	95 °C/ 15 min, followed by 35 cycles of 95 °C/ 30 s, 55 °C/ 30 s and 72 °C/ 1 min, with a final extension of 72 °C/ 7 min	[97]
			bgiR (reverse: 5′-CTCGACGAGCTTCGTGTT-3′)			
	*gdh*	786	Ghd1 (forward: 5′-TTCCGTRTYCAGTACAACTC-3′)	[53]	94 °C/ 2 min, followed by 35 cycles of 94 °C/ 30 s, 50 °C/ 30 s and 72 °C/ 1 min, with a final extension of 72 °C/ 7 min	[53]
			Gdh2 (reverse: 5′-ACCTCGTTCTGRGTGGCGCA-3′)			
		530	Gdh3 (forward: 5′-ATGACYGAGCTYCAGAGGCACGT-3′)		Secondary amplification was achieved employing identical PCR conditions to those used in the primary PCR	
			Gdh4 (reverse: 5′-GTGGCGCARGGCATGATGCA-3′)			
	SSU	497	Gia2029 (forward: 5′-AAGTGTGGTGCAGACGGACTC-3′)	[98]	94 °C/ 4 min, followed by 35 cycles of 96 °C/ 45 s, 55 °C/ 30 s and 72 °C/ 45 s, with a final extension of 72 °C/ 4 min	[98]
			Gia2150c (reverse: 5′-CTGCTGCCGTCCTTGGATGT-3′)			
		292	RH11 (forward: 5′-CATCCGGTCGATCCTGCC-3′)	[99]	94 °C/ 4 min, followed by 35 cycles of 96 °C/ 45 s, 59 °C/ 30 s and 72 °C/ 45 s, with a final extension of 72 °C/ 4 min	
			RH4 (reverse: 5′-AGTCGAACCCTGATTCTCCGCCAGG-3′)			
*Entamoeba*	SSU		JVC (forward: 5′-GTTGATCCTGCCAGTATTATATG-3′)	[100]	95 °C/ 5 min, followed by 40 cycles of 95 °C/ 30 s, 57 °C/ 30 s and 72 °C/ 1 min, with a final extension of 72 °C/ 4 min	[100]
			DSPR2 (reverse: 5′-CACTATTGGAGCTGGAATTAC-3′)			

Visualisation of PCR amplicons was achieved on 1.5% *w*/*v* agarose in 1× TBE (Tris, boric acid, ethylenediaminetetraacetic acid [EDTA] buffer) gel stained with SafeView Nucleic Acid Stain (Novel Biological Solutions, Huntingdon, UK). In brief, 5 μl of each amplicon was mixed with 1 μl of 6× DNA Loading Dye (ThermoFisher Scientific) and then subjected to electrophoresis at 50 V/45 min using TBE buffer (0.89 M Tris base, 0.89 M boric acid, 0.5 M EDTA; Sigma-Aldrich). A GeneRuler Low Range DNA Ladder (ThermoFisher Scientific) was included on each gel for size comparison purposes. All PCR amplicons were purified using a QIAquick® PCR Purification Kit (Qiagen), per the manufacturer’s instructions. Purified amplicons were subjected to cycle sequencing reactions using ABI Ready Reaction Mix (BigDye® Terminator v3.1 chemistry; Applied Biosystems, Foster City, USA) and the same primers employed for PCR (separately), followed by direct automated sequencing at GATC Biotech, Cologne, Germany. Comparison with corresponding electropherograms verified sequence quality and consensus sequences were constructed using the software package CLC Main Workbench v.6.9.1 (CLC bio, Aarhus, Denmark).

Basic Local Alignment Search Tool analyses (BLAST®: http://blast.ncbi.nlm.nih.gov/Blast.cgi) determined the sequence similarity of genetic data determined herein (GenBank Accession nos. KY658103–KY658190; Additional file 1: Table S1). Phylogenetic analysis was used to visualise relationships among *Entamoeba* sequence types defined here and those of 17 recognised species and 11 published ribosomal lineages, because of criteria defined by Jacob et al. [13]. Sequences were aligned using the software MUSCLE version 3.7 [15, 16] with ClustalW sequence weighting and UPGMA clustering for iterations 1 and 2. The resultant alignment was adjusted manually using the software BioEdit [17]. Phylogenetic analysis was conducted by Bayesian inference (BI) using Monte Carlo Markov Chain analysis in MrBayes 3.1.2 [18, 19]. The likelihood parameters set for BI analysis were based on the Akaike Information Criteria corrected for small sample sizes (AICc) [20] in jModelTest2 [21]. For the SSU data, we employed the general time-reversible model of evolution with a gamma distribution (GTR + Γ). Posterior probabilities (pp) were calculated via 1000,000 generations, utilising four simultaneous tree-building chains, with every 100th tree saved. At this point, the standard deviation of split frequencies was < 0.01, and the potential scale reduction factor approached 1. A 50% majority rule consensus tree was constructed based on the final 75% of trees generated by BI.

## Results

*Cryptosporidium*, *Giardia* and *Entamoeba* were detected in individual faecal samples from mountain gorillas, humans, and livestock from in and around BINP, while *Cyclospora* was not detected in any of the samples analysed (see Table 3). PCR detected three concurrent infections: one cattle from Kanyamisinga had *Giardia* (KY658189) and *Entamoeba* (KY658126); one goat from Mukono Church of Uganda had *Cryptosporidium* (KY658104) and *Entamoeba* (KY658147); and, a gorilla from South, Group Nkuringo had *Cryptosporidium* (KY658103) and *Entamoeba* (KY658168).

**Table 3:** The numbers of hosts test-positive for species of *Cryptosporidium*, *Giardia*, *Entamoeba* and *Cyclospora*, and the number of mixed infections

Host species	No. of samples examined	No. of positives (prevalence)						
		*Cryptosporidium parvum*	*Giardia duodenalis*	*Entamoeba bovis* ^a^	*Entamoeba coli* ^a^	*Entamoeba hartmanni* ^a^	*Cyclospora*	No. of mixed infections
*Gorilla beringei beringei*	68	1 (1.5%)			5 (7.4%)	33 (48.5%)		1 (*Cryptosporidium parvum* and *Entamoeba hartmanni*)
*Bos taurus*	45		2 (4.4%)	36 (80%)				1 (*Giardia duodenalis* AII and *Entamoeba bovis*)
*Capra hircus*	57	1 (1.7%)		34 (60%)				1 (*Cryptosporidium parvum* and *Entamoeba bovis*)
*Homo sapiens*	33		3 (9.1%)		3 (9.1%)			
Totals	203	2 (1.0%)	5 (2.5%)	70	8 (3.9%)	33 (16.3%)	0	3

^a^Based on the criteria of Jacob et al. [13], it is ‘technically’ not possible to classify *Entamoeba* genetic types to the level of species having amplified < 80% of SSU gene. However, based on initial sequence comparisons, and our phylogenetic analysis, we interpret sequence data with caution and classify *Entamoeba* samples as variants of *E. bovis*, *E. coli* or *E. hartmanni*

### Appraisal of sequence data, parasite identity and prevalence of infection

#### *Cryptosporidium*

We conducted sequence analyses of all *gp60* and SSU amplicons (*n* = 20 and 1, respectively) following PCR. These analyses identified amplicons from two of 20 (10%) faecal DNA samples to represent species and genotypes of *Cryptosporidium*. One sample was characterised at *gp60* (KY658103) and the other at SSU (KY658104); no sample was successfully characterised at both genes. The remaining 18 amplicons were identified as false positives (various bacteria) and are not considered further.

Comparison of the unique *gp60* and SSU sequence types determined herein with information available in the GenBank database inferred *Cryptosporidium parvum* from 1.5% of 68 gorillas (based on *gp60*) and 1.7% of 57 goats (based on SSU) tested. For the single individual faecal sample test positive in PCR for *gp60*, we characterised this isolate as genotype IId, subgenotype IIdA23G2, using the system of nomenclature proposed previously [22, 23].

#### *Giardia*

Sequencing of all *tpi* (*n* = 47), *gdh* (*n* = 24), *bg* (*n* = 3), and SSU (*n* = 5) test-positive PCR amplicons from 47 samples (nine from gorillas, 15 from humans and 23 from livestock) identified five samples to contain *Giardia* isolates representing a single genetic assemblage (A, B or E) of *G*. *duodenalis.* No sample represented mixed assemblage populations based on direct sequence comparisons. The remaining 42 samples were identified as false positive ‘bacteria’ or failed to sequence. For the five mono-assemblage samples, our analyses defined two distinct genotypes for *tpi* (represented by KY658189 and KY658190) and SSU (KY658186 and KY658187, and KY658188), and three genotypes for *gdh* and *bg* (KY658183–KY658185 and KY658180–KY658182, respectively). Comparison of these ten sequence types with information available in GenBank inferred *G. duodenalis* assemblage A in one of three (33%) individual faecal samples from humans from Buhoma and one of 15 (6.6%) cattle from Kanyamisinga; *G. duodenalis* assemblage B was inferred from two of 27 (7.4%) individual faecal samples from humans from Bujengwe Parish; and, *G. duodenalis* assemblage E was inferred from one of 12 (8.3%) individual faecal samples from cattle from Mukono, Church of Uganda. *Giardia* was not detected in samples from mountain gorillas or goats by PCR.

#### *Entamoeba*

Sequence analyses of all SSU amplicons (*n* = 111) identified the same number of faecal DNA samples to represent species/genotypes of *Entamoeba*. Comparison of the 74 unique SSU sequence types determined herein with information available in the GenBank database inferred the sequences with GenBank accession numbers KY658141 and KY658143 were identical to FN666250 and FN666252, respectively, for *Entamoeba bovis*, from 7.0% of 57 goats tested.

A further 72 new sequence types (GenBank accession nos. KY658105–KY658140, KY658142, KY658144–KY658179) were recorded during the present investigation. Two sequence types (KY658179 and KY658178), which differed by one and three nucleotides (1% over 630 bp) from a sequence of *E. coli* (FR686364), were each recorded in 3.0% of 33 human samples tested. A third sequence type (KY658177), which was 26 nucleotides different (4% over 631 bp) from a second *E. coli* sequence (AB4444953), was also recorded from 3.0% of humans tested. This same sequence type (KY658157) was detected in 1.5% of 68 gorillas tested, and is the only instance where a sequence type was shared between/among species. In the remaining 37 gorilla samples that tested positive for *Entamoeba*, four sequence types (KY658172, KY658155–KY658157) from five individuals, which are 20–27 nucleotides different (3–5% over 580 bp) from a sequence of *E. coli* (AB4444953), were recorded in 7.4% of 68 gorillas tested. Among these four sequence types, there are 2–7 nucleotide differences. The remaining 32 gorilla faecal samples contained *Entamoeba* samples with 19 different sequence types (KY658154, KY658158–KY658171, KY658173–KY658176) that differed from each other by 1–18 nucleotide differences (0.2–3.3% over 539 bp), and are 6–16 nucleotides different (1–3% over 539 bp) from a sequence of *E. hartmanni* (AF149907), originally reported from humans. From cattle, 29 sequence types (KY658105–KY658133) were defined from 36 individual faecal samples. These sequences differed by 1–51 nucleotides (0.2–9.4% over 545 bp) from each other, and by 15–27, 1–17, and 4–13 nucleotides (3–5%, 1–3%, and 1–2% over 545 bp) from three sequences of *E. bovis* (FN666249–FN666251, respectively), originally reported from cattle (FN666249) and sheep (FN666250 and FN666251). Finally, from goats, 18 new sequence types (KY658134–KY658153) were defined from 30 individual faecal samples, which differed by 1–53 nucleotides (0.2–9.7% over 546 bp) from each other. These sequence data are 4–7, 14–34, and 2–6 nucleotides different (1%, 3–6%, and 1% over 546 bp) from three sequences of *E. bovis* (FN666250–FN666252, respectively), originally reported from sheep (FN666250 and FN666251) and caribou (FN666252).

**Fig. 1:**
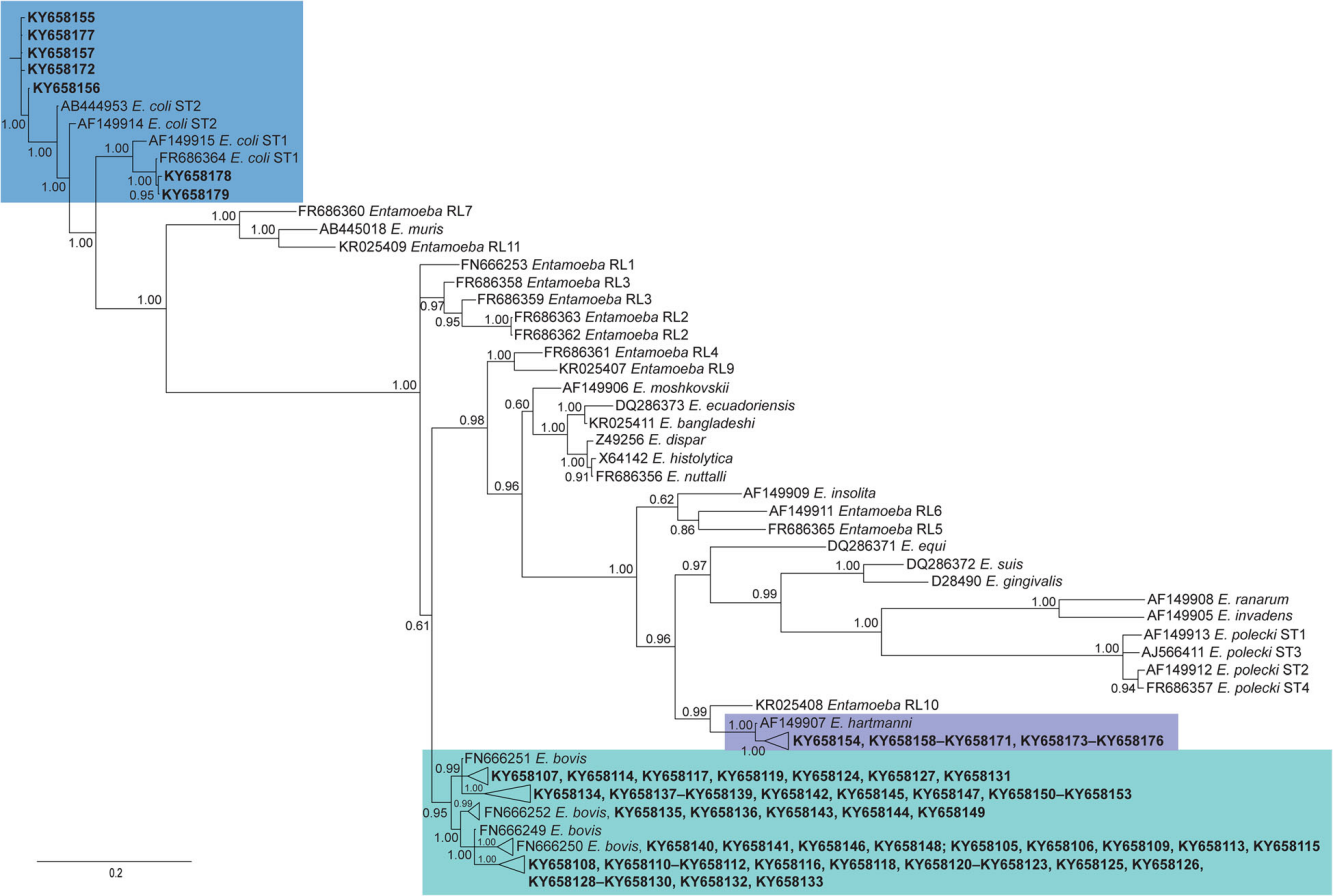
The relationships between species and ribosomal/conditional lineages of *Entamoeba* inferred from SSU sequence data following analysis by Bayesian inference (BI). Posterior probabilities are indicated at all major nodes. New sequences generated here shown in bold

The novel *Entamoeba* sequences defined here were aligned with 39 reference sequences obtained from GenBank. These reference sequences represented 17 recognised species and 11 published ribosomal lineages. All sequences were aligned across 611 positions. Phylogenetic analyses consistently grouped all 74 SSU sequence types with publicly available reference sequences representing *E. coli*, *E. bovis* and *E. hartmanni* (see Fig. 1), with strong support (pp = 0.95–1.00), to the exclusion of all sequences representing other recognised *Entamoeba* species, their subtypes, and ribosomal lineages.

#### Cyclospora

Sequencing of all amplicons (six and 30 from humans and mountain gorilla samples, respectively) identified that none of the samples tested contained *Cyclospora* DNA detectable by PCR. Based on sequence comparisons with data available on GenBank, genetic data determined herein indicated that amplicons were the result of the amplification of SSU from passerine *Eimeria* species (data not shown).

## Discussion

The present study genetically classified *Cryptosporidium*, *Giardia*, and *Entamoeba* in individual faecal samples from three potential host groups in and around BINP. Our systematic molecular analysis categorised all samples by comparison with reference data available in the GenBank database. The data provide no clear evidence for multiple inter-species transmission cycles (i.e. protists with the same sequence types shared among mountain gorillas, humans, or livestock). A fourth, *Cyclospora*, appears absent, or was below the limit of detection, from the 203 samples tested. The only sequence derived from multiple host groups was amplified from DNA extracted from an individual faecal sample from a human (fr. Buhoma) and a gorilla (fr. South, Group Bushaho) (*c*.10 km apart based on geographical coordinates; see Table 1). Comparison of this sequence type with public data indicated it was from an *Entamoeba* sample, which was 26 nucleotides or 4% different, over 631 bp, from an *E. coli* sequence (AB4444953). In contrast to these findings, previous epidemiological studies, on a broad range of pathogens, have frequently inferred cross-species transmission to be likely in the case of *Cryptosporidium* [24], *Giardia* [25], *Escherichia coli* [26] and *Encephalitozoon intestinalis* [27] in Uganda and Tanzania. However, in most cases, the prevalence of infection was higher than the 1.4%, 2.4%, and 54.7% for *Cryptosporidium*, *Giardia* and *Entamoeba*, respectively, determined here. Differences in prevalence of infection may be associated with the times of year samples were collected or differences in local habitat. Alternatively, these findings may reflect changes in Park management practices on faecal contamination of the region by local inhabitants, researchers, tourists, and Park Wardens, and proximity to agricultural land.

Using our PCR-based approach, we genetically characterised seven samples, which were assigned to *Cryptosporidium parvum*, and to the genetic assemblages A, B and E of *G*. *duodenalis*. *Cryptosporidium parvum* has an extremely broad host distribution making it the greatest zoonotic risk. It is also a cause of economic losses associated with bovine cryptosporidiosis [28]. Despite detecting *C. parvum* here, it was not found in humans or cattle, but from a gorilla and a goat. The low prevalence (1.4 and 1.7%, respectively, or 1% in all livestock) detected here may suggest a relatively low risk of transmission to humans and other mammals in/around BINP. Nonetheless, previous studies in this area have detected *C. parvum* from 11% of 100 gorillas [29], 38% of 50 cattle [30] and 8% of 62 humans [31] tested. In addition, in regions < 200 km from BINP, Salyer et al. [24] found the prevalence of infection in humans, non-human primates (NHP) and livestock as high as 32.4%, 11.1%, and 2.2%, respectively. These discrepancies highlight the need for further investigations of the presence and distribution of *Cryptosporidium* genotypes. Not only are these investigations essential to determine the potential significance of different host groups as sources, reservoirs, and amplifiers of *Cryptosporidium*, but also to establish which *C. parvum* transmission cycles occur naturally (e.g. human-to-human, animal-to-human and vice versa, and animal-to-animal) [32]. Understanding the underlying forces behind host-parasite relationships is important, particularly in areas such as BINP that are surrounded by extreme ecological imbalances (i.e. high human and livestock densities, both with low-quality health services), as a reduction in habitat can lead to changes in host density that result in alterations to host-parasite dynamics.

Of the two *Cryptosporidium* sequence types described here, only one has been reported previously. The single SSU sequence type (KY658104) appears to be distributed globally, having been recorded previously in animal hosts other than goats globally (e.g. [33–35]). While this sequence type has not been reported from humans, it has been characterised from cattle [34] and buffalo (Maurya PS. et al., unpublished), both of which are important livestock species. Given (i) the impoverished economic status of the human population surrounding BINP and the reliance on livestock animals to reduce chronic malnutrition, increase food security, and generate an alternate source of income, (ii) the economic losses that may result from bovine cryptosporidiosis, and (iii) the limited genetic information for parasites from herds in the region, ‘tracking’ *Cryptosporidium* spatially and temporally in Uganda is necessary for the future prevention and control of disease.

Based on *gp60* data, a single sequence was characterised as genotype IId of *C. parvum*, subgenotype IIdA23G2 (KY658103). This subgenotype and sequence type are, to the best of our knowledge, both new records. The genotype is also a new record for Uganda. The IId genotype has been reported from humans (e.g. [36–40]) and livestock animals (e.g. [41–50]), globally. Recently, genotype IId has been recorded in NHP from China [51], albeit with a different subgenotype (IIdA15G2R1). Consequently, genotype IId, along with IIa, is considered one of the two zoonotic subtype groups of *C. parvum* [52]. The presence of this genotype highlights the potential occurrence of zoonotic transmission in the region; however, further studies are needed to confirm transmission patterns of this genotype in the region.

From the five samples that tested positive in PCR for *G. duodenalis*, we did not find polymorphic nucleotide positions in *tpi*, *gdh*, or *bg* sequence types as has been reported previously (e.g. [53, 54]). Also in contrast to previous multilocus genotyping studies (e.g. [55, 56]), there was no discrepancy in assemblage assignment among the four genes for the five samples examined herein, albeit variable amplification success among genes prohibited phylogenetic analysis of a concatenated dataset. The sample characterised and assigned to assemblage A, sub-assemblage AII, based on sequence data from *gdh*, *bg* and SSU (KY658185, KY658181 and KY658188, respectively), and recorded here from a human, grouped with *G. duodenalis* genotypes reported from humans (e.g. [57–64]), wild and domestic animals (including cattle) (e.g. [65–68]) and water samples (e.g. [69, 70]) from around the world. Samples from a human assigned here to assemblage B, subassemblage BIV, based on sequence data from *gdh* and SSU (KY658183 and KY658187, respectively), grouped with *G. duodenalis* genotypes reported from humans from Australia, Brazil, the Netherlands and USA [71–73], and wild and domestic animals (including NHP) [74–77], globally. The second sample assigned to assemblage B, subassemblage BIV, based on phylogenetic analysis of sequence data from *bg* and SSU (KY658180 and KY658186, respectively), grouped with *G. duodenalis* SSU genotypes reported from humans from Australia, the Netherlands and USA [73, 78] and wild and domestic animals (including NHP) from Spain and Colombia [76, 77], globally. The corresponding *bg* sequence is new. Samples from cattle assigned to assemblage E on the basis of genetic data from *tpi*, *gdh* and *bg* (KY658190, KY658184 and KY658182, respectively), grouped with *tpi* and *gdh* genotypes reported previously from cattle in Bangladesh, China and USA [67, 79, 80], and NHP from China [51]. The corresponding *bg* sequence is new, as is the *tpi* AII sequence type (KY658189) recorded here from cattle.

*Giardia duodenalis* responsible for the human disease is most commonly linked to assemblages A and B [81, 82], while samples from livestock are typically linked to E. Of the subassemblages/genotypes described, AII, BIII, and BIV were considered specific to humans (see [83]). Nonetheless, multiple, related genotypes within each of these two assemblages have been detected in a range of animals (see [80, 84–86]). Our findings are consistent with this. Although it is not possible to illustrate the direct sharing of protist species among host groups using identical sequences, our finding of the *G*. *duodenalis* sub-assemblage AII in both a human and cattle indicates the potential for human-to-livestock transmission (based on published data; i.e. [87]).

The remaining samples positive in PCR were phylogenetically closest to *Entamoeba*. Our results showed an overall prevalence of 54.7% for *Entamoeba* sequence types from the 203 faecal samples, and 9.1%, 55.9%, 80.0% and 60.0% in human, mountain gorilla, cattle and goat faecal samples (or 68.6% of livestock faecal samples), respectively. The overall prevalence in mountain gorillas is comparable (55.9% in 68 samples *vs* 48.5% in 70 samples) to figures previously reported from Rwanda [88], despite the authors using microscopic examination and centrifugal flotation techniques. Although not the preferred method of identification [13], Sleeman et al. [88] also characterised *Entamoeba* samples to the level of species based on cyst and trophozoite morphology, detecting *E. coli*, *E. hartmanni* and *E. histolytica* in 20%, 27% and 1.4% of samples tested. Here, despite using what is the more sensitive/specific technique for the detection and classification of protists in faecal samples, we are ‘technically’ unable to classify all 74 sequence types to the level of species based on the criteria of Jacob et al. [13], having amplified < 80% of SSU gene (i.e. 539–580 of *c.*1400 nt). However, based on initial sequence comparisons, and our phylogenetic analysis (see Fig. 1), which includes reference sequences representing 17 recognised species and 11 published ribosomal lineages, we interpret 72 new sequence types with caution and classify them as variants (rather than species/subtypes/ribosomal lineages/conditional lineages) of *E. bovis*, *E. coli* or *E. hartmanni*. The two remaining sequence types were detected from faecal samples from goats (KY658141 and KY658143) from Kanyamisinga and Aidah-Rugira (respectively) and were 100% identical to two sequences for *E. bovis* (FN666250 and FN666252 from sheep and caribou, respectively) [89] over 546 nucleotides. Again, we use caution in this interpretation. Regardless, just two sequence types were shared among host groups, and (typically) these species are not considered pathogenic.

In addition to detecting the target parasites, many samples positive in PCR and sent for sequencing returned results linking amplicons to bacteria or passerine *Eimeria* (data not shown). This finding highlights the advantages of using phenetic-based approaches, i.e. RFLP, single-strand conformation polymorphism (SSCP), or restriction endonuclease fingerprinting-SSCP, coupled with sequencing to screen large numbers of samples by detecting point mutations, group samples by profile and only sequence representative amplicons. Mutation scanning can, therefore, be a sensitive and powerful tool for the direct analysis of subtle genetic variation within and among populations of protists isolated from animals and the environment.

## Conclusions

The present study has provided a snapshot of the occurrence and genetic make-up of *Cryptosporidium*, *Giardia*, *Entamoeba* and *Cyclospora* in mammals in BINP. The genetic analyses indicated that 54.6% of the 203 samples contained *Cryptosporidium*, *Giardia* or *Entamoeba* that matched species, genotypes or assemblages with the potential to infect humans, mountain gorillas, and livestock species. In addition, 76 new sequence records were identified. As nothing is known about the zoonotic/zooanthroponotic potential of protist samples with these sequences, future work should focus on wider epidemiological investigations of these genetic types together with continued surveillance of protists in humans, other mammals, the environment, and water in this highly impoverished area.

## Additional file

Additional file 1: Table S1. Sequence data determined in this study, together with epidemiological information. (DOCX 15 kb)

## Abbreviations

AIC: Akaike information criteria
*bg*: beta-giardin gene
BI: Bayesian inference
BINP: Bwindi Impenetrable National Park
CTPH: Conservation through Public Health
EDTA: Ethylenediaminetetraacetic acid
*gdh*: Glutamate dehydrogenase gene
gDNA: Total genomic DNA
*gp60*: 60 kDa glycoprotein gene
ID: Infectious diseases
MCMC: Monte Carlo Markov chain
NHP: Non-human primate
PBS: Phosphate buffered saline
Pp: Posterior probability
RFLP: Restriction fragment length polymorphism
RVC: Royal Veterinary College
SSCP: Single-strand conformation polymorphism
SSU: Small subunit of the ribosomal DNA
TBE: Tris, boric acid, ethylenediaminetetraacetic acid buffer
*tpi*: triosephosphate isomerase gene

## References

[1] Messenger AM, Barnes AN, Gray GC (2014). Reverse zoonotic disease transmission (zooanthroponosis): a systematic review of seldom-documented human biological threats to animals. PLoS One.

[2] de Graaf DC, Vanopdenbosch E, Ortega-Mora LM, Abbassi H, Peeters JE (1999). A review of the importance of cryptosporidiosis in farm animals. Int J Parasitol.

[3] Leroy EM, Rouquet P, Formenty P, Souquière S, Kilbourne A, Froment JM (2004). Multiple Ebola virus transmission events and rapid decline of central African wildlife. Science.

[4] Cully JF, Barnes AM, Quan TJ, Maupin G (1997). Dynamics of plague in a Gunnison's prairie dog colony complex from New Mexico. J Wildl Dis.

[5] Ginsberg JR, Mace GM, Albon S (1995). Local extinction in a small and declining population: wild dogs in the Serengeti. Proc R Soc Lond Ser B.

[6] Robbins M, Gray M, Kümpel N, Lanjouw A, Maisels F, Mugisha A, et al. *Gorilla beringei* ssp. *beringei*. The IUCN Red List of Threatened Species 2008: e.T39999A10292321 2008 [cited 2016 07/11/2016].

[7] Gray M, Fawcett K, Basabose A, Cranfield M, Vigilant L, Roy J, et al. Virunga Massif mountain gorilla census −2010 summary. report. 2011.

[8] Robbins MM, Roy J, Wright E, Kato R, Kabano P, Basabose A (2011). Bwindi mountain gorilla census 2011- summary of results.

[9] Cranfield MR (2008). Mountain gorilla research: the risk of disease transmission relative to the benefit from the perspective of ecosystem health. Am J Primatol.

[10] Guerrera W, Sleeman JM, Ssebide BJ, Pace LB, Ichinose TY, Reif JS (2003). Medical survey of the local human population to determine possible health risks to the mountain gorillas of Bwindi impenetrable Forest National Park. Uganda Int J Primatol.

[11] Jex AR, Smith HV, Monis PT, Campbell BE, Gasser RB (2008). *Cryptosporidium -* biotechnological advances in the detection, diagnosis and analysis of genetic variation. Biotechnol Adv.

[12] Monis PT, Andrews RH, Mayrhofer G, Ey PL (2003). Genetic diversity within the morphological species *Giardia intestinalis* and its relationship to host origin. Infect Genet Evol.

[13] Jacob AS, Busby EJ, Levy AD, Komm N, Clark CG (2016). Expanding the *Entamoeba* universe: new hosts yield novel ribosomal lineages. J Eukaryot Microbiol.

[14] Ortega YR, Sanchez R (2010). Update on *Cyclospora cayetanensis*, a food-borne and waterborne parasite. Clin Microbiol Rev.

[15] Edgar RC (2004). MUSCLE: a multiple sequence alignment method with reduced time and space complexity. BMC Bioinformatics.

[16] Edgar RC (2004). MUSCLE: multiple sequence alignment with high accuracy and high throughput. Nucleic Acids Res.

[17] Hall TA (1999). Bio edit: a user-friendly biological sequence alignment editor and analysis program for windows 95/98/NT. Nucleic Acids Symp Ser.

[18] Huelsenbeck JP, Ronquist F (2001). MRBAYES: Bayesian inference of phylogenetic trees. Bioinformatics.

[19] Ronquist F, Huelsenbeck JP (2003). MrBayes 3: Bayesian phylogenetic inference under mixed models. Bioinformatics.

[20] Hurvich CM, Tsai A (1989). Regression and time series model selection in small samples. Biometrika.

[21] Posada G, Crandall KA (1998). Modeltest: testing the model of DNA substitution. Bioinformatics.

[22] Sulaiman IM, Hira PR, Zhou L, Al-Ali FM, Al-Shelahi FA, Shweiki HM (2005). Unique endemicity of cryptosporidiosis in children in Kuwait. J Clin Microbiol.

[23] Chalmers RM, Robinson G, Elwin K, Hadfield SJ, Xiao L, Ryan UM (2009). *Cryptosporidium* sp. rabbit genotype, a newly identified human pathogen. Emerg Infect Dis.

[24] Salyer SJ, Gillespie TR, Rwego IB, Chapman CA, Goldberg TL (2012). Epidemiology and molecular relationships of *Cryptosporidium* spp. in people, primates, and livestock from western Uganda. PLoS Negl Trop Dis.

[25] Johnston AR, Gillespie TR, Rwego IB, McLachlan TL, Kent AD, Goldberg TL (2010). Molecular epidemiology of cross-species *Giardia duodenalis* transmission in western Uganda. PLoS Negl Trop Dis.

[26] Rwego IB, Gillespie TR, Isabirye-Basuta G, Goldberg TL (2008). High rates of *Escherichia coli* transmission between livestock and humans in rural Uganda. J Clin Microbiol.

[27] Graczyk TK, Bosco-Nizeyi J, da Silva AJ, Moura IN, Pieniazek NJ, Cranfield MR (2002). A single genotype of *Encephalitozoon intestinalis* infects free-ranging gorillas and people sharing their habitats in Uganda. Parasitol Res.

[28] Santín M, Trout JM, Fayer R (2008). A longitudinal study of cryptosporidiosis in dairy cattle from birth to 2 years of age. Vet Parasitol.

[29] Graczyk TK, DaSilva AJ, Cranfield MR, Nizeyi JB, Kalema GR, Pieniazek NJ (2001). *Cryptosporidium parvum* genotype 2 infections in free-ranging mountain gorillas (*Gorilla gorilla beringei*) of the Bwindi impenetrable National Park Uganda. Parasitol Res.

[30] Nizeyi JB, Cranfield MR, Graczyk TK (2002). Cattle near the Bwindi impenetrable National Park, Uganda, as a reservoir of *Cryptosporidium parvum* and *Giardia duodenalis* for local community and free-ranging gorillas. Parasitol Res.

[31] Nizeyi JB, Sebunya D, Dasilva AJ, Cranfield MR, Pieniazek NJ, Graczyk TK (2002). Cryptosporidiosis in people sharing habitats with free-ranging mountain gorillas (*Gorilla gorilla beringei*) Uganda. Am J Trop Med Hyg.

[32] Smith HV, Cacciò SM, Cook N, Nichols RA, Tait A (2007). *Cryptosporidium* and *Giardia* as foodborne zoonoses. Vet Parasitol.

[33] Feng Y, Alderisio KA, Yang W, Blancero LA, Kuhne WG, Nadareski CA (2007). *Cryptosporidium* genotypes in wildlife from a New York watershed. Appl Environ Microbiol.

[34] Karanis P, Eiji T, Palomino L, Boonrod K, Plutzer J, Ongerth J (2010). First description of *Cryptosporidium bovis* in Japan and diagnosis and genotyping of *Cryptosporidium* spp. in diarrheic pre-weaned calves in Hokkaido. Vet Parasitol.

[35] Twomey DF, Barlow AM, Bell S, Chalmers RM, Elwin K, Giles M (2008). Cryptosporidiosis in two alpaca (*Lama pacos*) holdings in the south-west of England. Vet J.

[36] Ng J, MacKenzie B, Ryan U (2010). Longitudinal multi-locus molecular characterisation of sporadic Australian human clinical cases of cryptosporidiosis from 2005 to 2008. Exp Parasitol.

[37] Abe N, Matsubayashi M, Kimata I, Iseki M (2006). Subgenotype analysis of *Cryptosporidium parvum* isolates from humans and animals in Japan using the 60-kDa glycoprotein gene sequences. Parasitol Res.

[38] Adamu H, Petros B, Zhang G, Kassa H, Amer S, Ye J (2014). Distribution and clinical manifestations of *Cryptosporidium* species and subtypes in HIV/AIDS patients in Ethiopia. PLoS Negl Trop Dis.

[39] Zintl A, Ezzaty-Mirashemi M, Chalmers RM, Elwin K, Mulcahy G, Lucy FE (2011). Longitudinal and spatial distribution of GP60 subtypes in human cryptosporidiosis cases in Ireland. Epidemiol Infect.

[40] O'Brien E, McInnes L, Ryan U (2008). *Cryptosporidium* GP60 genotypes from humans and domesticated animals in Australia North America and Europe. Exp Parasitol.

[41] Muhid A, Robertson I, Ng J, Ryan U (2011). Prevalence of and management factors contributing to *Cryptosporidium* sp. infection in pre-weaned and post-weaned calves in Johor, Malaysia. Exp Parasitol.

[42] Amer S, Zidan S, Adamu H, Ye J, Roellig D, Xiao L (2013). Prevalence and characterization of *Cryptosporidium* spp. in dairy cattle in Nile River delta provinces, Egypt. Exp Parasitol.

[43] Li F, Wang H, Zhang Z, Li J, Wang C, Zhao J (2016). Prevalence and molecular characterization of *Cryptosporidium* spp. and *Giardia duodenalis* in dairy cattle in Beijing, China. Vet Parasitol.

[44] Broglia A, Reckinger S, Cacciò SM, Nöckler K (2008). Distribution of *Cryptosporidium parvum* subtypes in calves in Germany. Vet Parasitol.

[45] Tzanidakis N, Sotiraki S, Claerebout E, Ehsan A, Voutzourakis N, Kostopoulou D (2014). Occurrence and molecular characterization of *Giardia duodenalis* and *Cryptosporidium* spp. in sheep and goats reared under dairy husbandry systems in Greece. Parasite.

[46] Geurden T, Thomas P, Casaert S, Vercruysse J, Claerebout E. Prevalence and molecular characterisation of *Cryptosporidium* and *Giardia* in lambs and goat kids in Belgium. Vet Parasitol 2008; 155 (1–2):142–5. 10.1016/j.vetpar.2008.05.002 18565678

[47] Helmy YA, Krücken J, Nöckler K, von Samson-Himmelstjerna G, Zessin KH (2013). Molecular epidemiology of *Cryptosporidium* in livestock animals and humans in the Ismailia province of Egypt. Vet Parasitol.

[48] Qi M, Cai J, Wang R, Li J, Jian F, Huang J (2015). Molecular characterization of *Cryptosporidium* spp. and *Giardia duodenalis* from yaks in the central western region of China. BMC Microbiol.

[49] Galuppi R, Piva S, Castagnetti C, Iacono E, Tanel S, Pallaver F (2015). Epidemiological survey on *Cryptosporidium* in an equine Perinatology unit. Vet Parasitol.

[50] Vieira PM, Mederle N, Lobo ML, Imre K, Mederle O, Xiao L, et al. Molecular characterisation of *Cryptosporidium* (Apicomplexa) in children and cattle in Romania. Folia Parasitol (Praha). 2015;62:002. 10.14411/fp.2015.002 25960546

[51] Du SZ, Zhao GH, Shao JF, Fang YQ, Tian GR, Zhang LX (2015). *Cryptosporidium* spp., *Giardia intestinalis*, and *Enterocytozoon bieneusi* in captive non-human primates in Qinling Mountains. Korean J Parasitol.

[52] Plutzer J, Karanis P (2009). Genetic polymorphism in *Cryptosporidium* species: an update. Vet Parasitol.

[53] Cacciò SM, Beck R, Lalle M, Marinculic A, Pozio E (2008). Multilocus genotyping of *Giardia duodenalis* reveals striking differences between assemblages a and B. Int J Parasitol.

[54] Lebbad M, Mattsson JG, Christensson B, Ljungström B, Backhans A, Andersson JO (2010). From mouse to moose: multi locus genotyping of *Giardia* isolates from various animal species. Vet Parasitol.

[55] Read CM, Monis PT, Thompson RC (2004). Discrimination of all genotypes of *Giardia duodenalis* at the glutamate dehydrogenase locus using PCR-RFLP. Infect Genet Evol.

[56] Levecke B, Geldhof P, Claerebout E, Dorny P, Vercammen F, Cacciò SM (2009). Molecular characterisation of *Giardia duodenalis* in captive non-human primates reveals mixed assemblage a and B infections and novel polymorphisms. Int J Parasitol.

[57] Wegayehu T, Karim MR, Li J, Adamu H, Erko B, Zhang L (2016). Multilocus genotyping of *Giardia duodenalis* isolates from children in Oromia special zone, central Ethiopia. BMC Microbiol.

[58] de Lucio A, Martinez-Ruiz R, Merino FJ, Bailo B, Aguilera M, Fuentes I (2015). Molecular genotyping of *Giardia duodenalis* isolates from symptomatic individuals attending two major public hospitals in Madrid Spain. PLoS One.

[59] Ferreira FS, Centeno-Lima S, Gomes J, Rosa F, Rosado V, Parreira R (2012). Molecular characterization of *Giardia duodenalis* in children from the Cufada lagoon Natural Park Guinea-Bissau. Parasitol Res.

[60] Laishram S, Kannan A, Rajendran P, Kang G, Ajjampur SS (2012). Mixed *Giardia duodenalis* assemblage infections in children and adults in South India. Epidemiol Infect.

[61] Kosuwin R, Putaporntip C, Pattanawong U, Jongwutiwes S (2010). Clonal diversity in *Giardia duodenalis* isolates from Thailand: evidences for intragenic recombination and purifying selection at the beta giardin locus. Gene.

[62] Bonhomme J, Le Goff L, Lemée V, Gargala G, Ballet JJ, Favennec L (2011). Limitations of *tpi* and *bg* genes sub-genotyping for characterization of human *Giardia duodenalis* isolates. Parasitol Int.

[63] Cacciò SM, De Giacomo M, Pozio E (2002). Sequence analysis of the *β-giardin* gene and development of a polymerase chain reaction-restriction fragment length polymorphism assay to genotype *Giardia duodenalis* cysts from human faecal samples. Int J Parasitol.

[64] Yong TS, Park SJ, Hwang UW, Yang HW, Lee KW, Min DY (2000). Genotyping of *Giardia lamblia* isolates from humans in China and Korea using ribosomal DNA sequences. J Parasitol.

[65] Gómez-Couso H, Ortega-Mora LM, Aguado-Martínez A, Rosadio-Alcántara R, Maturrano-Hernández L, Luna-Espinoza L (2012). Presence and molecular characterisation of *Giardia* and *Cryptosporidium* in alpacas (*Vicugna pacos*) from Peru. Vet Parasitol.

[66] Geurden T, Goossens E, Levecke B, Vercammen F, Vercruysse J, Claerebout E (2009). Occurrence and molecular characterization of *Cryptosporidium* and *Giardia* in captive wild ruminants in Belgium. J Zoo Wildl Med..

[67] Wang H, Zhao G, Chen G, Jian F, Zhang S, Feng C (2014). Multilocus genotyping of *Giardia duodenalis* in dairy cattle in Henan China. PLoS One.

[68] Santín M, Dargatz D, Fayer R (2012). Prevalence of *Giardia duodenalis* assemblages in weaned cattle on cow-calf operations in the United States. Vet Parasitol.

[69] Fernandes LN, de Souza PP, de Araújo RS, Razzolini MT, Soares RM, Sato MI (2011). Detection of assemblages a and B of *Giardia duodenalis* in water and sewage from São Paulo state Brazil. J Water Health.

[70] Koloren Z, Seferoğlu O, Karanis P (2016). Occurency of *Giardia duodenalis* assemblages in river water sources of Black Sea Turkey. Acta Trop.

[71] Souza SL, Gennari SM, Richtzenhain LJ, Pena HF, Funada MR, Cortez A (2007). Molecular identification of *Giardia duodenalis* isolates from humans, dogs, cats and cattle from the state of São Paulo, Brazil, by sequence analysis of fragments of glutamate dehydrogenase (*gdh*) coding gene. Vet Parasitol.

[72] Lasek-Nesselquist E, Welch DM, Thompson RC, Steuart RF, Sogin ML (2009). Genetic exchange within and between assemblages of *Giardia duodenalis*. J Eukaryot Microbiol.

[73] van Keulen H, Homan WL, Erlandsen SL, Jarroll EL (1995). A three nucleotide signature sequence in small subunit rRNA divides human *Giardia* in two different genotypes. J Eukaryot Microbiol.

[74] Prystajecky N, Tsui CK, Hsiao WW, Uyaguari-Diaz MI, Ho J, Tang P (2015). *Giardia* spp. are commonly found in mixed assemblages in surface water, as revealed by molecular and whole-genome characterization. Appl Environ Microbiol.

[75] Soares RM, de Souza SL, Silveira LH, Funada MR, Richtzenhain LJ, Gennari SM. Genotyping of potentially zoonotic *Giardia duodenalis* from exotic and wild animals kept in captivity in Brazil. Vet Parasitol 2011; 180 (3–4):344–8. 10.1016/j.vetpar.2011.03.049 21530084

[76] Martinez-Diaz RA, Sansano-Maestre J, Martinez-Herrero Mdel C, Ponce-Gordo F, Gomez-Munoz MT (2011). Occurrence and genetic characterization of *Giardia duodenalis* from captive nonhuman primates by multi-locus sequence analysis. Parasitol Res.

[77] Santín M, Cortés Vecino JA, Fayer R (2013). A large scale molecular study of *Giardia duodenalis* in horses from Colombia. Vet Parasitol.

[78] Thompson RC, Hopkins RM, Homan WL (2000). Nomenclature and genetic groupings of *Giardia* infecting mammals. Parasitol Today.

[79] Ehsan AM, Geurden T, Casaert S, Parvin SM, Islam TM, Ahmed UM (2015). Assessment of zoonotic transmission of *Giardia* and *Cryptosporidium* between cattle and humans in rural villages in Bangladesh. PLoS One.

[80] Feng Y, Ortega Y, Cama V, Terrel J, Xiao L (2008). High intragenotypic diversity of *Giardia duodenalis* in dairy cattle on three farms. Parasitol Res.

[81] Mayrhofer G, Andrews RH, Ey PL, Chilton NB. Division of *Giardia* isolates from humans into two genetically distinct assemblages by electrophoretic analysis of enzymes encoded at 27 loci and comparison with *Giardia muris*. Parasitology 1995; 111(1):11–17. 10.1017/s0031182000064556 7609985

[82] Monis PT, Mayrhofer G, Andrews RH, Homan WL, Limper L, Ey PL (1996). Molecular genetic analysis of *Giardia intestinalis* isolates at the glutamate dehydrogenase locus. Parasitology.

[83] Cacciò SM, Ryan U (2008). Molecular epidemiology of giardiasis. Mol Biochem Parasitol.

[84] Sulaiman IM, Fayer R, Bern C, Gilman RH, Trout JM, Schantz PM (2003). Triosephosphate isomerase gene characterization and potential zoonotic transmission of *Giardia duodenalis*. Emerg Infect Dis.

[85] Traub RJ, Monis PT, Robertson I, Irwin P, Mencke N, Thompson RC (2004). Epidemiological and molecular evidence supports the zoonotic transmission of *Giardia* among humans and dogs living in the same community. Parasitology.

[86] Lasek-Nesselquist E, Bogomolni AL, Gast RJ, Welch DM, Ellis JC, Sogin ML (2008). Molecular characterization of *Giardia intestinalis* haplotypes in marine animals: variation and zoonotic potential. Dis Aquat Org.

[87] Monis PT, Cacciò SM, Thompson RC (2009). Variation in *Giardia*: towards a taxonomic revision of the genus. Trends Parasitol.

[88] Sleeman JM, Meader LL, Mudakikwa AB, Foster JW, Patton S (2000). Gastrointestinal parasites of mountain gorillas (*Gorilla gorilla beringei*) in the Parc national des Volcans Rwanda. J Zoo Wildl Med.

[89] Stensvold CR, Lebbad M, Clark CG (2010). Genetic characterisation of uninucleated cyst-producing *Entamoeba* spp. from ruminants. Int J Parasitol.

[90] Li G, Xiao S, Zhou R, Li W, Wadeh H (2007). Molecular characterization of *Cyclospora*-like organism from dairy cattle. Parasitol Res.

[91] Sulaiman IM, Ortega Y, Simpson S, Kerdahi K (2014). Genetic characterization of human-pathogenic *Cyclospora cayetanensis* parasites from three endemic regions at the 18S ribosomal RNA locus. Infect Genet Evol.

[92] Xiao L, Morgan UM, Limor J, Escalante A, Arrowood M, Shulaw W (1999). Genetic diversity within *Cryptosporidium parvum* and related *Cryptosporidium* species. Appl Environ Microbiol.

[93] Morgan UM, Constantine CC, Forbes DA, Thompson RC (1997). Differentiation between human and animal isolates of *Cryptosporidium parvum* using rDNA sequencing and direct PCR analysis. J Parasitol.

[94] Peng MM, Matos O, Gatei W, Das P, Stantic-Pavlinic M, Bern C (2001). A comparison of *Cryptosporidium* subgenotypes from several geographic regions. J Eukaryot Microbiol.

[95] Glaberman S, Moore JE, Lowery CJ, Chalmers RM, Sulaiman I, Elwin K (2002). Three drinking-water-associated cryptosporidiosis outbreaks Northern Ireland. Emerg Infect Dis.

[96] Alves M, Xiao L, Sulaiman I, Lal AA, Matos O, Antunes F (2003). Subgenotype analysis of *Cryptosporidium* isolates from humans, cattle, and zoo ruminants in Portugal. J Clin Microbiol.

[97] Lalle M, Pozio E, Capelli G, Bruschi F, Crotti D, Cacciò SM (2005). Genetic heterogeneity at the β-giardin locus among human and animal isolates of *Giardia duodenalis* and identification of potentially zoonotic subgenotypes. Int J Parasitol.

[98] Appelbee AJ, Frederick LM, Heitman TL, Olson ME (2003). Prevalence and genotyping of *Giardia duodenalis* from beef calves in Alberta Canada. Vet Parasitol.

[99] Hopkins RM, Meloni BP, Groth DM, Wetherall JD, Reynoldson JA, Thompson RC (1997). Ribosomal RNA sequencing reveals differences between the genotypes of *Giardia* isolates recovered from humans and dogs living in the same locality. J Parasitol.

[100] Santos HLC, Bandyopadhyay K, Bandea R, Peralta RHS, Peralta JM, Da Silva AJ (2013). LUMINEX®: a new technology for the simultaneous identification of five *Entamoeba* spp. commonly found in human stools. Parasit Vectors.

