# Supplementary material for: Molecular characterisation of protist parasites in human-habituated mountain gorillas (Gorilla beringei beringei), humans and livestock, from Bwindi impenetrable National Park, Uganda
Source: Parasit Vectors. 2017 Jul 18;10:340. doi: 10.1186/s13071-017-2283-5 (PMC5516388; doi:10.1186/s13071-017-2283-5)
Supplement: Additional file 1: Table S1. — Sequence data determined in this study, together with epidemiological information. (DOCX 15 kb) [file 13071_2017_2283_MOESM1_ESM.docx]

**Additional file 1: Table S1.** Sequence data determined in this study, together with epidemiological information

| **Species** | **Subgenotype/genetic assemblage** | **Host source** | **GenBank Accession nos.** | **Gene** |
| --- | --- | --- | --- | --- |
| *Cryptosporidium parvum* | na | Goat (*Capra hircus*) | KY658104 | SSU |
|  | IIdA23G2 | Mountain gorilla (*Gorilla beringei beringei)* | KY658103 | *gp60* |
| *Giardia duodenalis* | A | Cattle (*Bos taurus*) | KY658189 | *tpi* |
|  | E | Cattle | KY658190 | *tpi* |
|  | A | Human (*Homo sapiens*) | KY658185 | *gdh* |
|  | B | Human | KY658183 | *gdh* |
|  | E | Cattle | KY658184 | *gdh* |
|  | A | Human | KY658181 | *bg* |
|  | B | Human | KY658180 | *bg* |
|  | E | Cattle | KY658182 | *bg* |
|  | A | Human | KY658188 | SSU |
|  | B | Human | KY658186, KY658187 | SSU |
| *Entamoeba bovis* | na | Cattle | KY658105–KY658133 | SSU |
|  | na | Goat | KY658134–KY658153 | SSU |
| *Entamoeba coli* | na | Human | KY658177–KY658179 | SSU |
|  | na | Mountain gorilla | KY658155–KY658157, KY658172 | SSU |
| *Entamoeba hartmanni* | na | Mountain gorilla | KY658154, KY658158–KY658171, KY658173–KY658176 | SSU |

*Abbreviation*: na, not applicable.
